# Evaluation of Adverse Drug Reaction Profile of Drugs Used as First-Line Antiretroviral Therapy

**DOI:** 10.1155/2018/8095609

**Published:** 2018-08-02

**Authors:** Mukta N. Chowta, Priyanka Kamath, John T. Ramapuram, K. Ashok Shenoy, Sanjay Hadigal

**Affiliations:** ^1^Department of Pharmacology, Kasturba Medical College, Mangalore, Manipal Academy of Higher Education, India; ^2^Department of General Medicine, Kasturba Medical College, Mangalore Manipal Academy of Higher Education, India; ^3^Medical Affairs, Mylan, Bengaluru, India

## Abstract

**Background and Objectives:**

The objective was to study the adverse drug reaction (ADR) profile in HIV patients receiving first-line antiretroviral therapy.

**Methods:**

This was a prospective, observational study that included 171 HIV patients with a follow-up at six months. Demographic details, medical history, details of HIV infection including most recent CD4 count, details of antiretroviral therapy, and other concomitant medication were recorded. Adverse drug reactions were elicited by reviewing patient records and also by interviewing the patient/attendants directly.

**Results:**

171 patients completed the study out of which 88 (51.5%) were males and 83 (48.5%) were females. The study subjects included HIV-positive, treatment naïve patients who were started on treatment regimens recommended by the NACO guidelines. The ADRs observed were a fall in haemoglobin or absolute anaemia in response to zidovudine, nonspecific symptoms like headache, and a nonspecific feeling of being unwell in response to tenofovir, stavudine, and efavirenz; dyslipidaemia, pancreatitis, peripheral neuropathy, and lactic acidosis in response to stavudine; generalised rash in response to nevirapine and one case of nephrotoxicity to efavirenz. Majority of the ADRs satisfied the ‘probable' category (60.1%), and the rest were “possible”. ADRs to zidovudine and nevirapine superseded all others.

**Interpretation and Conclusion:**

Gastrointestinal effects were the most commonly observed group of ADRs, with nausea being the most common ADR, the others being gastritis and diarrhoea. The other ADRs included rash, hepatotoxicity, blood dyscrasias like anaemia, neutropenia, and thrombocytopenia, and fatigue. Few cases of lactic acidosis, peripheral neuropathy, headache, lipoatrophy, and pancreatitis were reported.

## 1. Introduction

HIV-AIDS, first recognized in the United States in the summer of 1981, was considered an incurable and terminal infection, till the development and availability of effective antiretroviral drugs. Development of these drugs and in particular their use as* combination* therapy have significantly improved the outcome in a patient infected with HIV [1].

The* Consolidated Guidelines on the use of Antiretroviral Drugs for Treating and Preventing HIV Infection, Recommendations for a Public Health Approach*, were published by the World Health Organization (WHO) in 2016. These guidelines outline a detailed schema for diagnosis, treatment of HIV, and the related opportunistic infections, what to expect during the first six months of treatment, monitoring response to treatment, dealing with antiretroviral drug toxicities and substitutions, managing comorbidities, HIV prevention including pre- and postexposure prophylaxis, switch of antiretroviral regimen, and the general care of the HIV-positive individual. These guidelines also encompass details on how to provide the various services in different age groups and vulnerable groups including adolescents [2]. Based on these guidelines, different countries have adapted the same to suit their healthcare systems and policies.

In India, the National AIDS Control Organization (NACO) publishes guidelines regularly, outlining the steps for diagnosis and treatment of HIV infection, the most recent ones being those published in 2013. According to these guidelines, the ideal time to start ART is before the patient presents with an opportunistic infection [3, 4]. However, the current WHO recommendation with moderate-quality evidence states that ART can be initiated in all adults living with HIV, regardless of the WHO clinical stage, at any CD4 cell count, and as a priority, ART* has to be* initiated in* all* adults with severe or advanced HIV clinical disease (WHO clinical stage 3 or 4) and adults with CD4 count ≤350 cells/mm^3^ [2]. This is being implemented in our country as well, and ART is now being initiated regardless of the WHO stage and CD4 counts, based on the patients' informed decision to start ART.

The aim of treatment is to bring about viral suppression to levels as low as possible for a time period as long as possible. More often than not, once started the therapy has to be continued for life. Many of these drugs are known to cause adverse effects, which can range from being mild to even life threatening [2, 4]. A strict adherence to the prescribed regimen is vital for the success of therapy and to bring about a reduction of the viral load.

Many studies have been conducted in Western and African population to study the adverse effect profile to ART, but such studies are scanty in the Indian population. In addition, results from different studies have raised more questions, paving the way for further research in HIV therapeutics in order to optimize treatment with the available resources.

This study was designed to study the toxicity profile towards the commonly used first-line antiretroviral drugs (ART).

## 2. Methods

The study was conducted at a tertiary care teaching hospital in South India; the study population consisted of HIV-positive subjects of either gender who were on ART. It was conducted after approval from the institutional ethics committee was obtained. It was an observational longitudinal study, with a follow-up at 6 months, and the sample size was calculated to be 171 HIV-positive patients who were on ART.

HIV-positive patients aged more than 18 years on ART or those who were to be initiated on ART, whose baseline CD4 cell counts were available, were included in the study. Severely ill patients whose life expectancy was less than one week at recruitment, patients on antitubercular treatment/other drugs known to cause renal or hepatic toxicity at the time of recruitment, patients with history of alcohol abuse at the time of recruitment, and patients who were not compliant with ART were excluded from the study.

Data was collected from the patient case sheet after each clinic visit and also by interviewing the patient directly. The patients' demographic details were recorded at the baseline visit. Demographic data collected included age, gender, height, weight, and socioeconomic status. Any other new symptoms and/or any other new diagnoses were also recorded.

CD4 cell count was recorded at baseline and at six months. Other laboratory investigations that were collected at baseline included blood counts, liver function tests, renal function tests, blood sugar, and fasting lipid profile (whatever available). Any clinically indicated additional investigations and results were also recorded.

Details regarding HIV infection were also collected with respect to mode of infection, duration of illness, clinical signs and symptoms, WHO staging of the disease, presence opportunistic infections. Details on antiretroviral treatment collected included type of regimen that was initiated, date on which it was started, switch-over to any other regimen, and the reason for the switch. ART regimen was categorized as follows, in accordance with the NACO guidelines [4]:

  Regimen I: AZT +3TC +NVP
  Regimen II: d4T + 3TC+NVP
  Regimen III: AZT +3TC +EFV
  Regimen IV: d4T + 3TC + EFV
  Regimen V: TDF + 3TC + NVP
  Regimen VI: TDF + 3TC + EFV
  Regimen VII: Second line regimen containing protease inhibitor
  Regimen VIII: Any other regimen

(AZT = Zidovudine, 3TC = Lamivudine, NVP = Nevirapine, EFV = Efavirenz, TDF = Tenofovir, d4T = Stavudine)

Details of other concomitant medications received by the patients were also collected with the dose, duration, and indication.

### 2.1. ADR Monitoring

WHO definition of ADR was used (any response to a medicine which is noxious and unintended and which occurs at doses normally used in man). ADRs were evaluated using standard clinical signs and symptoms. WHO-UMC causality scale was used to assess the causality and the ADRs were classified into certain, probable/likely, possible, unlikely, conditional/unclassified, and unassessable/unclassifiable, with* certain *being defined as an event or laboratory test abnormality with plausible time relationship to drug intake which could not be explained by disease or other drugs,* probable/likely *being defined as an event or laboratory test abnormality with a reasonable time relationship to drug intake, which was unlikely to be attributed to disease or other drugs;* possible* being defined as an event or laboratory test with a reasonable time relationship to drug intake but which could also be explained by disease or other drugs;* unlikely* being defined as an event or laboratory test abnormality with a time to drug intake that made a relationship improbable (but not impossible);* conditional/unclassified* being defined as an event or laboratory test abnormality for which more data was needed for proper assessment; and* unassessable/unclassifiable* being defined as a report suggesting an adverse reaction but which could not be judged because information was insufficient or contradictory and data could not be supplemented or verified [5].

Adverse drug reactions were elicited by reviewing patient records and also by interviewing patient/attendant directly whenever possible. The details of ADRs collected included suspected drugs involved, treatment given for ADRs, and the outcome. Naranjo's algorithm was also used for causality assessment [6].

### 2.2. Statistical Analysis

The collected data was entered in MS-Excel and analysis was done using SPSS, version 16.0. For qualitative data, statistical test Chi square was done, and wherever appropriate, Fischer Exact test was done. Continuous data was analysed by using Student's “t” test. p-value <0.05 was taken as statistically significant. Baseline characteristics were compared by applying the Student's t test and Levene's test for equality of variances.

## 3. Results

A total of 171 patients completed the study of 6 months duration. Data from these patients was available for analysis. The study subjects included HIV-positive, treatment naïve patients who were started on ART regimens recommended by the NACO guidelines. The CD4 cell counts were assessed at baseline and at 6 months. Routine blood investigations were done at baseline and thereafter whenever warranted.

All the mentioned baseline characteristics were matched between genders, except for haemoglobin. On applying Levene's test for equality of variances, the difference in haemoglobin between genders was found to be statistically significant (equal variances assumed, F = 26.535, p < 0.001). A positive correlation was seen between baseline haemoglobin and baseline CD4 cell count (r = 0.308, p < 0.001). A positive correlation was also observed between the baseline body mass index (BMI) and baseline haemoglobin (r = 0.281, p < 0.001). [Table 1]

Six different treatment regimens were instituted in patients, based on various factors such as baseline haemoglobin and availability of drugs at the time of institution of therapy. Some difference was seen in the institution of various treatment regimens between gender, but this was not statistically significant (p=0.109). [Table 2]

Out of 171 patients, 79 of them experienced at least one ADR, of which 34 were female patients and 45 were male patients. The causality of ADRs was assessed using World Health Organization (WHO) ADR probability scale and the Naranjo's algorithm [5, 6]. The ADRs observed were a fall in haemoglobin or absolute anaemia in response to zidovudine, nonspecific symptoms like headache and a general feeling of being unwell in response to tenofovir, stavudine, and efavirenz; dyslipidemia, pancreatitis, peripheral neuropathy, and lactic acidosis in response to stavudine; generalised rash in response to nevirapine and one case of nephrotoxicity to efavirenz. Majority of the ADRs satisfied the “probable” category (60.1%), and the rest were “possible”. Since rechallenge was not done on the development of an ADR, the certainty of the ADR could not be proved; none of the ADRs were “unlikely” either [Table 3(a)].

Zidovudine and nevirapine were the two drugs towards which the maximum number of ADRs was seen. Out of 171 patients, 109 were on a zidovudine containing regimen, and 26 of them developed an ADR to zidovudine, of which 7 were females and 19 were males. The ADRs included either absolute anaemia or a fall in haemoglobin. In such patients, since the baseline haemoglobin was high, the fall in haemoglobin was not large enough to cause overt anaemia. Out of these 26 patients, 8 patients also developed thrombocytopenia, and 9 patients developed neutropenia. On the development of ADR with zidovudine, it was replaced by another NRTI, like tenofovir or stavudine. Two patients developed an ADR to both zidovudine and nevirapine.

Reaction to nevirapine was seen in 26 patients out of the 135 patients receiving nevirapine containing regimen; of these 15 were female patients and 11 were males. Generalised papular rash was observed in all these patients and hepatotoxicity in eight of these patients. Nonspecific symptoms like nausea and vomiting were also seen.

At the time of initiation of this study, stavudine as a part of first-line ART was slowly being phased out, due to intolerable side effects that it had been proven to cause over the years. Hence, there were only 39 patients out of 171 who were on a stavudine containing regimen. Of these, 12 patients developed an ADR towards stavudine (six males, six females). The symptoms ranged from nonspecific symptoms like nausea to raised lactate levels, dyslipidemia, and lipoatrophy. Three cases of lactic acidosis and one case of pancreatitis was seen. Two cases of peripheral neuropathy attributable to stavudine were also seen.

**Figure 1:**
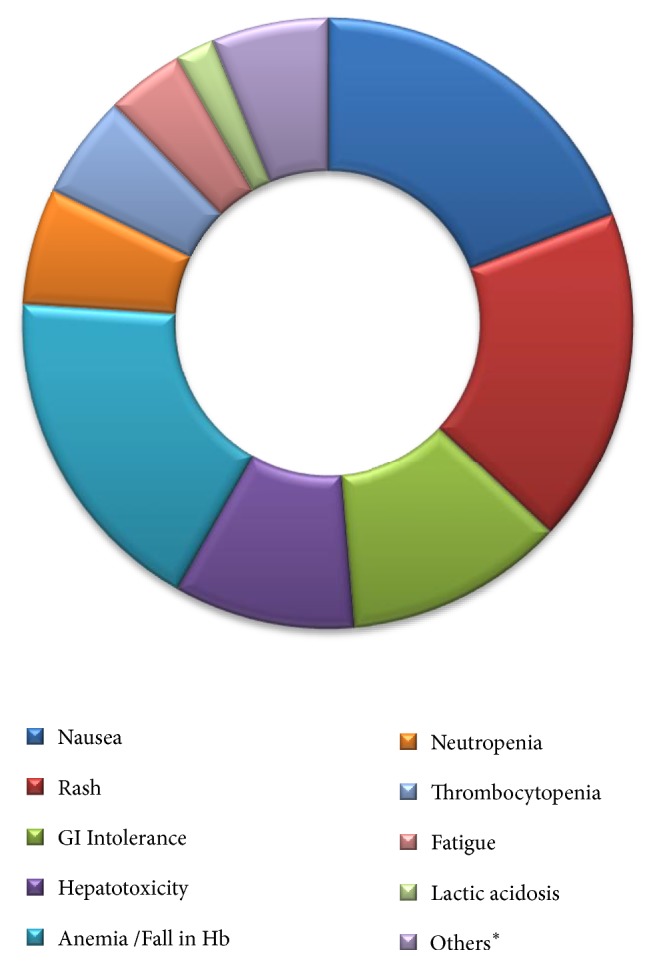
Adverse drug reactions observed. ^*∗*^Others include dyslipidemia, peripheral neuropathy, headache with insomnia, nephrotoxicity, pancreatitis, and lipoatrophy.

Thirty-six patients were initially started on an efavirenz containing regimen, of which nine patients developed an ADR. Six of them were male patients and three were female. There were general symptoms like insomnia, headache, and nausea in these patients. Hepatotoxicity occurred in six of them with raised transaminase levels. Efavirenz was also substituted for nevirapine in patients who developed an ADR towards nevirapine [Table 3(b), Figure 1].

Twenty-three patients out of 171 were started on a tenofovir containing regimen. Tenofovir was also given instead of zidovudine in those patients who developed a reaction in response to zidovudine. Four patients out of these developed an ADR towards tenofovir of which two were male patients and two female. Of these, one male patient developed nephrotoxicity with proteinuria. Generalised weakness was seen in the other three patients.

All patients had an adherence of >95% to the prescribed regimen. On the development of any ADR or intolerance towards the medication, spontaneous reporting was done and suitable steps like change in regimen or counselling regarding medications or both were done as indicated.

## 4. Discussion

The aim of the present study was to study the pattern of the occurrence of adverse drug reactions to the first-line antiretroviral drugs. All baseline characteristics between genders were matched and no significant difference was seen, except in haemoglobin. As already known, haemoglobin was found to be significantly lower in female patients in comparison to males at baseline and this has been previously reported in another study [7]. This physiological difference has been attributed to higher levels of tissue oxygenation for a given red cell mass and more efficient tissue red cell delivery in females [8].

The results from prior studies regarding the incidence of ADRs have been varying. Various studies have reported a prevalence of ADRs to ART as low as 19.5% to as high as 86% [9–11]. Another study put the prevalence at 39.7% [12]. Based on these studies, the sample size for this study was calculated considering an incidence of ADR as 50%. In our study, out of 171 patients, 79 of them experienced at least one ADR, which puts the incidence of ADRs at 46.19%. Zidovudine and nevirapine were the most common drugs to which ADRs developed, and this was seen in an earlier study as well. Majority of the ADRs satisfied the “probable” category (60.1%), which was also reported in the same study [13].

ART is the treatment of choice in HIV patients; these drugs once started have to be continued lifelong. Hence, toxicity to these drugs is a very important issue in the management of HIV-infected patients, as this determines the compliance of patients to the therapeutic regimen that has been initiated. The most common regimen used was a combination of zidovudine, lamivudine, and nevirapine, accounting for 52.6% of the patients, followed by a combination of stavudine, lamivudine, and nevirapine (18.71%). This was in accordance with the guidelines being followed in the period during which the study was conducted [4]. However, the current recommended regimen of choice is a combination of tenofovir, lamivudine, and efavirenz, which accounted for only 5.84% in our study [2]. This change in the regimen of choice could partly be due to increased incidence of adverse reactions to the drugs used. Hence, it reflects the importance of long-term studies to assess the effect of ART, and the implications of the same in bringing about policy change.

In our study, ADRs to zidovudine and nevirapine superseded all others. The reason for this could probably be that these two drugs were the most frequently employed first-line drugs among the various regimens employed. Hence the number of ADRs observed towards these two drugs was also higher. Gastrointestinal intolerance, anaemia, thrombocytopenia, and dermatological adverse effects were the commonly observed ADRs, along with others like hepatotoxicity, dyslipidemia, pancreatitis, lactic acidosis, and peripheral neuropathy.

Nevirapine has been known to cause dermatological reactions like itching and rash, which are a part of the hypersensitivity spectrum. This has been studied previously in animal models and it was seen that 12-hydroxylation of nevirapine producing the metabolite 12-OH-nevirapine causes the rash; 12-OH-nevirapine when administered was also seen to cause a rash [14].

Zidovudine is known to cause bone marrow suppression which in turn can cause anaemia and thrombocytopenia. WHO definition of anaemia was used, wherein a haemoglobin level of less than 12 gm/dl is considered as anaemia in females, and less than 13 gm/dl is considered anaemia in males [15]. In our study, the total number of patients developing an ADR to zidovudine consisted of more males than females, which could be explained as males had a higher baseline value of haemoglobin.

Lactic acidosis, peripheral neuropathy, and pancreatitis also were seen in our study. Patients who developed lactic acidosis presented with abdominal pain, nausea, and vomiting. Lactic acidosis and peripheral neuropathy are well-documented adverse effect of stavudine, with a previous study reporting high incidence of lactic acidosis in HIV-infected patients receiving stavudine as a single agent [16–18]. Pancreatitis is a sign of mitochondrial toxicity. The two important mitochondrial catabolic pathways which are implicated here are pyruvate oxidation by pyruvate dehydrogenase and fatty acid oxidation via *β*-oxidation. Hence, the metabolic pathway of pyruvic acid is shifted towards increased lactate production, and fatty acids are converted to triglycerides that accumulate in the hepatocyte cytoplasm. Not all patients develop lactic acidosis; this could be due to acquired deficiencies in riboflavin and thiamine cofactors required for oxidative phosphorylation, which may predispose to the development of lactic acidosis [3, 16]. Peripheral neuropathy was seen in two patients. It is known to occur to stavudine and didanosine; however in our study there were no patients who received didanosine, and hence it was attributed to be caused due to stavudine.

ADRs due to efavirenz were largely nonspecific, with some patients showing raised serum transaminase levels suggestive of hepatotoxicity. Efavirenz is known to be hepatotoxic, albeit not as much as nevirapine. A case report has also suggested that the risk of hepatotoxicity with efavirenz could be higher if used along with tenofovir [19, 20]. The most commonly reported ADR towards tenofovir is gastrointestinal effects; tenofovir induced nephrotoxicity is explained by mitochondrial DNA depletion which causes mitochondrial toxicity; other reactions seen towards other NRTIs are less common with tenofovir [21, 22]. In our study, patients who developed an ADR towards tenofovir presented with nonspecific symptoms, and one of them developed proteinuria which is a marker of nephrotoxicity.

Our study had certain limitations; baseline and sixth-month virological load were not done routinely unless indicated, and hence correlation between the virological response which can affect the response to therapy and the occurrence of ADRs was not possible. Also, the follow-up period was for six months only, and in order to draw a conclusion, longer follow-ups would be required. Since the patients included were treatment naïve, no patients on Protease Inhibitors were included, and hence the toxicity profile towards the same could not be studied.

## 5. Conclusion

Gastrointestinal effects were the most commonly observed group of ADRs, with nausea being the most common ADR, the others being gastritis and diarrhoea. The other ADRs also included rash, hepatotoxicity, blood dyscrasia like anaemia, neutropenia and thrombocytopenia, and fatigue. Few cases of lactic acidosis, peripheral neuropathy, headache, lipoatrophy, and pancreatitis were also reported.

## Figures and Tables

**Table 1:** Demographic characteristics at baseline.

Parameter	Female	Male	Total
Number (%)	83 (48.5)	88 (51.5)	171 (100)

Age	42.16 ± 9.50	43.79 ± 9.43	43.00 ± 9.47

BMI	19.95 ± 3.33	20.42 ± 3.34	20.19 ± 3.33

Haemoglobin	11.25 ± 1.49	12.43 ± 2.46*∗*	11.86 ± 2.12

CD4 count	245.04 ± 96.81	232.86 ± 94.79	238.77 ± 95.69

Figures expressed as mean ± SD; Student's t-test: *∗* = highly significant; p<0.001.

**Table 2:** Treatment regimens by gender.

Treatment Regimen	Gender		Total
	Female	Male	
Regimen I (AZT+3TC+NVP)	43	47	90

Regimen II (d4T+3TC+NVP)	19	13	32

Regimen III (AZT+3TC+EFV)	6	13	19

Regimen V (TDF+3TC+NVP)	7	6	13

Regimen VI (TDF+3TC+EFV)	7	3	10

Regimen IV (d4T+3TC+EFV)	1	6	7

Total	83	88	171

Chi-square test; AZT = Zidovudine, 3TC = Lamivudine, NVP = Nevirapine, EFV = Efavirenz, TDF = Tenofovir, and d4T = Stavudine.

**Table:** (a) Antiretroviral drugs and ADRs

	Antiretroviral drugs				
	Zidovudine	Nevirapine	Stavudine	Efavirenz	Tenofovir
Number of ADRs	28	28	12	9	4

Percentage	16.3%	16.3%	7.02%	5.26%	2.33%

**Table:** (b) Distribution of adverse drug reactions by gender

**ADR**	**Males**	**Females**	**Total**	**p value**
Nausea	12	16	28	0.408

Rash + Itching	11	15	26	0.39

Fall in Hb/Anemia	19	7	26	0.019*∗*

Gastrointestinal Intolerance	10	7	17	0.613

Hepatotoxicity	8	6	14	0.78

Neutropenia	3	6	9	0.318

Thrombocytopenia	3	5	8	0.486

Fatigue	2	4	6	0.43

Lactic acidosis	1	2	3	0.61

Dyslipidemia	1	1	2	1.00

Peripheral neuropathy	1	1	2	1.00

Headache+Insomnia	1	1	2	1.00

Nephrotoxicity	1	0	1	1.00

Pancreatitis	1	0	1	1.00

Lipoatrophy	0	1	1	0.48

Chi-square test; *∗*=significant.

## Data Availability

Data was collected from the tertiary care hospital attached to KMC Mangalore, Manipal Academy of Higher Education.
